# Under what conditions do climate-driven sex ratios enhance versus diminish population persistence?

**DOI:** 10.1002/ece3.1316

**Published:** 2014-11-19

**Authors:** Maria Boyle, Jim Hone, Lisa E Schwanz, Arthur Georges

**Affiliations:** 1 Institute for Applied Ecology, University of CanberraCanberra, ACT, 2601, Australia; 2 School of Biological, Earth and Environmental Sciences, University of New South WalesSydney, NSW, 2052, Australia

**Keywords:** TSD, GSD, reptiles, survival, sex ratio, male limitation, population dynamics

## Abstract

For many species of reptile, crucial demographic parameters such as embryonic survival and individual sex (male or female) depend on ambient temperature during incubation. While much has been made of the role of climate on offspring sex ratios in species with temperature-dependent sex determination (TSD), the impact of variable sex ratio on populations is likely to depend on how limiting male numbers are to female fecundity in female-biased populations, and whether a climatic effect on embryonic survival overwhelms or interacts with sex ratio. To examine the sensitivity of populations to these interacting factors, we developed a generalized model to explore the effects of embryonic survival, hatchling sex ratio, and the interaction between these, on population size and persistence while varying the levels of male limitation. Populations with TSD reached a greater maximum number of females compared to populations with GSD, although this was often associated with a narrower range of persistence. When survival depended on temperature, TSD populations persisted over a greater range of temperatures than GSD populations. This benefit of TSD was greatly reduced by even modest male limitation, indicating very strong importance of this largely unmeasured biologic factor. Finally, when males were not limiting, a steep relationship between sex ratio and temperature favoured population persistence across a wider range of climates compared to the shallower relationships. The opposite was true when males were limiting – shallow relationships between sex ratio and temperature allowed greater persistence. The results highlight that, if we are to predict the response of populations with TSD to climate change, it is imperative to 1) accurately quantify the extent to which male abundance limits female fecundity, and 2) measure how sex ratios and peak survival coincide over climate.

## Introduction

Reptiles with temperature-dependent sex determination (TSD) are considered to be particularly vulnerable to climate warming, owing to the production of biased primary sex ratios (sex ratios of offspring) (Fuentes et al. 2009, 2011; Hays et al. 2003; Hawkes et al. 2007, 2009; Mitchell et al. 2008; Wapstra et al. 2009; Witt et al. 2010; Patino-Martinez et al. 2012). Biased primary sex ratios may have detrimental effects on local population growth and persistence, although their quantitative impact has been poorly explored. Kallimanis (2010) argued that geographic ranges (and range expansion) are limited by poor population growth at the range boundary due to biased sex ratios, either toward males or females (Kallimanis 2010). In contrast, Freedberg and Taylor (2007) argue that population growth is enhanced by female-biased sex ratios.

The issue of climate-driven biases in offspring sex ratio is an immediate one. Janzen (1994) predicted that a 4°C temperature increase (relative to the present) may eventually eliminate males in a population of freshwater painted turtles (*Chrysemys picta*). In most TSD reptiles, females are produced at the higher temperatures. In contrast, tuatara (two species, *Sphenodon punctatus* and *Sphenodon guntheri*), a rare and taxonomically unique reptile endemic to New Zealand, has the uncommon pattern of TSD where males are produced at higher temperatures. In the tuatara, climate modeling predicts that 100% male hatchlings could be produced in less than 100 years, leading to local population extinctions by 2085 (Mitchell et al. 2010). While the projected risk of extinction in tuatara appears to be relatively low in the longer term, more recent estimates indicate that declines in adult and embryonic survival and in the proportion of female hatchlings, may contribute to an extinction “vortex,” resulting in more rapid local population extinctions (Grayson et al. 2014).

Notwithstanding that TSD taxa have survived climate warming and cooling over evolutionary timeframes (Mitchell and Janzen 2010), it remains unclear if TSD reptiles are able to respond quickly enough to contemporary human-induced climate warming through evolutionary compensatory mechanisms, or if they have scope to respond through phenotypic plasticity (Morjan 2003; Weishampel et al. 2004; Schwanz and Janzen 2008; Telemeco et al. 2009; Mitchell and Janzen 2010). Current evidence clearly indicates that we may expect many populations to produce increasingly biased sex ratios in the next few decades (Janzen 1994; Hawkes et al. 2007, 2009; Mitchell and Janzen 2010). Thus, elucidating the demographic impact of biased offspring sex ratios is a pressing scientific undertaking.

There remain many challenges to predicting the impact of air temperature on population size and persistence, largely due to a dearth of empirical data. The first is that we do not know much about the quantitative relationship between air temperature and offspring, or cohort sex ratio (CSR). In contrast, much is known of how individual or clutch sex ratio varies with incubation temperature (the TSD reaction norm) (Bull 1980; Janzen and Paukstis 1991). The former relationship can be modeled through CSR response curves, which describe the relationship between the proportion of hatchlings that develop as male and ambient air temperature (Schwanz et al. 2010). The CSR response curve has been described in only a limited number of studies (Hawkes et al. 2007; Wapstra et al. 2009; Schwanz et al. 2010), but may prove to be a useful predictive tool for local population growth, decline and extinction as climates warm.

The second challenge is that we do not know how dependent female fecundity is on the abundance of males. Assuming biased offspring sex ratios translate into biased adult sex ratios, males may become limiting as they become rare, leading to reduced egg production and threatening population persistence (Rankin and Kokko 2007). Studies of non-reptile species with female-biased sex ratios have found varying results on the effects of reduced numbers of males on female fecundity and population viability. For example, in the saiga antelope (*Saiga tatarica*) (Milner-Gulland et al. 2003), trophy hunting led to very female-biased adult sex ratios. As a consequence, females were unable to find mates, and fecundity and population viability declined considerably (Milner-Gulland et al. 2003). In contrast, in the butterfly *Hypolimnas bolina,* where the ratio of males to females is around 1 to 100, a very small numbers of males can fertilize large numbers of females successfully so that population viability is largely unaffected (Dyson and Hurst 2004).

There are parallels between our study, where sex ratios of reptile species with thermolabile sex are potentially skewed by climate change, and fisheries, where sex ratios are skewed by sex specific harvest, particularly in those species exhibiting sequential sexual phenotypes (protogynous) where male limitation can lead to population collapse (Alonzo and Mangel 2004). Male limitation has occurred under low male densities in gag fish (*Mycteroperca microlepsis*) (Heppell et al. 2006), California sheepshead fish (*Semicossyphus pulcher*) (Hamilton et al. 2007; Alonzo et al. 2008), and black sea bass (*Centropristis striata*) (Alonzo et al. 2008) where a natural female bias in the sex ratio has been exacerbated by male-biased fisheries harvesting.

Similarly, in crustaceans such as the blue crab (*Callinectes sapidus*) (Kendall et al. 2002; Carver et al. 2005) and snow crab (*Chionoecetes opilio)* (Sainte-Marie et al. 2008), fishing directed at large males may reduce the average size of males and male density, increasing female biases in the sex ratio. This in turn may affect the mating dynamics of the population, through the consistent production of smaller, less fecund males (smaller males produce less sperm in many species) and restricted mate choice for females, increasing male limitation, and potential reproductive failure (Kendall et al. 2002; Carver et al. 2005; Sainte-Marie et al. 2008).

Previous studies have demonstrated that fish populations of Atlantic silverside (*Menidia menidia*) may have the genetic resources to evolve rapidly as a response to sustained environmental pressure (harvesting of large males) that has skewed the sex ratio. After around 12 generations, the sex ratio shifted from skewed to even (Conover et al. 1992). Indeed, manipulated sex ratio skew can be a tool in the management of invasive pest species (Stelkens and Wedekind 2010).

The strength of male limitation on female fecundity is thought to be quite low in TSD populations (Broderick et al. 2000; Hawkes et al. 2009) due to males mating with multiple females (Broderick et al. 2001; Pearse et al. 2002) and sperm storage by females for up to 4 years (Pearse et al. 2002). In some lizards, multiple mating may reduce the effect of male limitation on female fecundity as climates warm (Uller and Olsson 2008). However, we know of no quantitative estimates of male limitation for any reptile populations.

The final challenge we wish to consider is the impact air temperature may have on embryonic survival in reptile species with both TSD and GSD. Most of embryonic development in oviparous reptiles occurs in the nest, and hence, embryonic survival is strongly linked with environmental temperatures (Georges et al. 2005). If temperatures rise (or fall) rapidly then embryonic survival will be adversely impacted unless changes in nesting behavior compensate for climate change (Girondot et al. 2004). Reduced embryonic survival is predicted as climates warm in reptiles with both TSD and GSD (Hawkes et al. 2007, 2009; Telemeco et al. 2013). Hawkes et al. (2007) predicted for loggerhead turtles (*Caretta caretta*) that a 6°C raise in air temperature across 100 years would result in 100% embryonic mortality.

For many reptiles, embryonic survival follows a “bell-shaped” (or approximately normal) distribution between the extreme temperatures of 17 and 40°C (Birchard 2004). In marine turtles, temperature ranges are reported as approximately 24–35°C (Yntema and Mrosovsky 1982; Hawkes et al. 2007), 22–32°C for the painted turtle (Schwarzkopf and Brooks 1987) and 20–30°C for the snapping turtle (Steyermark and Spotila 2001). Outside of turtles, the range of temperatures for embryonic survival has similarly been estimated as 18–25°C for the tuatara (*Sphenodon punctatus*; Thompson 1990), approximately 23–32°C across twenty species of Australian agamid lizards (Harlow 2004), and 25–37°C for a mound-nesting crocodilian (*Crocodylus porosus*; Webb and Cooper-Preston 1989). Embryonic or hatchling survival may also vary as a result of local adaptation to temperature (Weber et al. 2012). For example, in green turtle (*Chelonia mydas*) embryos, two different populations a few kilometers apart on Ascension Island (UK) display different thermal tolerance associated with different local sand temperatures (Weber et al. 2012).

It seems unavoidable that male limitation and temperature-dependent embryonic survival will interact with temperature-dependent sex ratios to influence population persistence. However, the nature and importance of that interaction are unknown and have not been examined before. For example, how sensitive is population persistence to variation in the strength of male limitation? What is the effect of varying the cohort sex ratio at temperatures where peak embryonic survival occurs? In this study, we develop generalized models of TSD and GSD populations to determine and compare how sex-determining mechanisms, juvenile survival, and male limitation interact with climate in influencing population persistence. Although quantitative predictions regarding the extent of population persistence in any particular species would require input of species-specific parameter values, our generalized model has the advantage of (1) providing insight into underlying processes that drive the population dynamics in these species and (2) yielding predictions that can be tested empirically and that can indicate prime targets for future empirical work. We focus on short-term ecological responses of reptile populations and not on evolutionary responses.

## Materials and Methods

### Model description

We used a generalized model of populations of females with GSD, GSD and TS (temperature-dependent embryonic survival), TSD and TSD and TS in a range of stable air temperatures. We explored the population outcomes across a range of empirically informed parameter values that we anticipate encompass most reptile species. The GSD scenarios served as null models so that we could separate the independent and interactive effects of temperature-dependent sex ratio and survival on persistence. We considered four CSR response curves (including GSD, see below), four temperature-dependent embryonic survival (TS) curves (including no temperature dependence, see below), and three levels of male limitation (none, moderate, and strong) to investigate the relative effects of TS, the CSR response curve, and the interaction between these and male limitation on population size and population persistence. Population persistence is defined as the range of temperatures at which populations exist with non-zero population size.

### Population size

We described density-dependent deterministic population growth using a logistic growth equation:


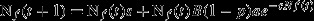
(1)

where *N*_f_(*t*) is the female population size at time t. The parameters in equations 1 (Table1) were for a long-lived animal consistent with average life expectancy for some reptiles of around 20 years (Congdon et al. 1994; Heppell 1998). Baseline embryonic survival (*a*) was density-dependent and the larger the value of *c*, the stronger the effect of density on embryonic survival. Age at first reproduction was set at 1 year (Lande 1988). Each time step is equivalent to 1 year.

**Table 1:** Parameters and associated values used in population equations 1 and 2 (NA = Not applicable; Temperature-dependent sex determination (TSD))

Parameter description	Symbol	Default value	Range of values (if applicable)	Source
Annual adult survival	s	0.95	NA	Congdon et al. (1994)
Baseline embryonic survival	a	0.015	0–0.015, depending on temperature	Heppell (1998)
Number of offspring (eggs laid) per adult female	B	*B*_max_ *,* 10	0–10, depending on male limitation	We chose the value 10 to produce a stable population (no male limitation)
Proportion of hatchlings that develop as male	*p*	0.5	TSD: 0–1, depending on temperature GSD: 0.5	Bull (1980)
Parameter for density dependence in embryonic survival	c	0.001	NA	We chose this value to produce a low effect of density dependence
Air temperature (°C)	T		16–35°C	Birchard (2004)

Equation 1 was re-arranged to estimate the population size of females at equilibrium by setting *N*_f_(*t + 1*) =  *N*_f_(*t*), and denoting 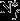 = K (carrying capacity) (Equation 2).


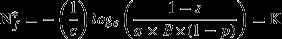
(2)

Parameters *a* (survival) and *p* (sex ratio) could depend on temperature and *B* could depend on *p*. We consider these parameters in turn below.

### Cohort sex ratio and temperature (*p*)

**Figure 1:**
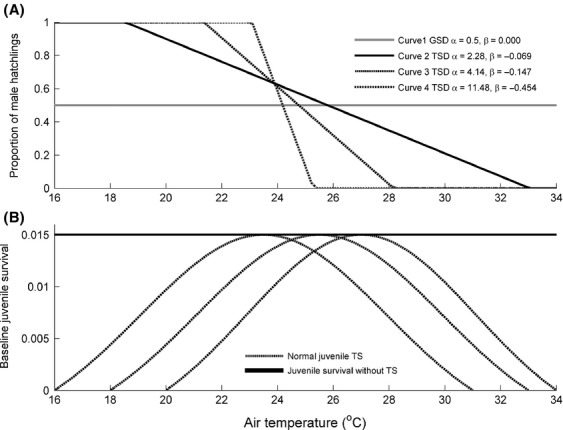
Cohort sex ratio (CSR) response curves and baseline embryonic survival curves. (A) CSR response curves for GSD (Curve 1). TSD (Curve 3) uses the regression equation parameters for the slope and intercept estimated from data on the painted turtle (Schwanz et al. 2010). TSD (Curves 2 and 4) use parameters for species with shallower and steeper slopes for CSR response curves, respectively. (B) Normal (dashed) distributions (TS curves) of baseline embryonic survival *a* distributed along temperature gradients 1, 2, and 3. Maximum baseline embryonic survival (*a*_max_ = 0.015) is denoted by the solid line.

The first CSR response curve was for GSD populations, where the offspring sex ratio was 0.5 for all air temperatures. Curve 1 (Fig.1A) represents GSD with corresponding parameters for the slope (*β * = 0.0) and intercept (*α * = 0.5). Because the CSR curve is likely to vary across TSD populations and is known for only a few species, we considered results for several different CSR response curves (Hawkes et al. 2007; Wapstra et al. 2009; Schwanz et al. 2010) (Table2, Fig.1A). CSR response Curve 2, the first TSD curve (Fig.1A) represents a species, such as the loggerhead turtle, with a shallow slope (*β * = −0.069) an intercept of *α * = 2.28. Curve 3 (Fig.1A) uses the slope observed for the painted turtle and snow skink (*β * = −0.147; Wapstra et al. 2009; Schwanz et al. 2010) and the painted turtle intercept (*α*  = 4.14). CSR response Curve 4 (Fig.1A) represents a species with a steeper slope (*β * = −0.454, *α * = 11.48), although no empirical examples of such a steep relationship are currently known. The curves cross at approximately 0.6 proportion male offspring because this was the sex ratio produced at the long-term average air temperature for painted turtles (Schwanz et al. 2010). Because we calculated population size for stable air temperatures, *p* did not fluctuate across years.

**Table 2:** Details of cohort sex ratio (CSR) response curves for the snow skink, painted turtle, and loggerhead turtle

Species	Slope (*β*)	Range of air temperatures with intermediate CSR	Source
Painted turtle (*Chrysemys picta*)	−0.147	21–27°C	Schwanz et al. (2010)
Snow skink (*Niveoscincus ocellatus*)	−0.147	17–18°C	Wapstra et al. (2009)
Loggerhead turtle (*Caretta caretta*)	−0.069	24–29°C	Hawkes et al. (2007)

### Embryonic survival and temperature (*a*)

In the absence of temperature effects on survival, embryonic survival (*a* e^− *c* Nf^) depended only on density, ranging in magnitude from the maximum baseline embryonic survival (*a * = * * 0.015, or 15 of every 1000 hatchlings survive) at the lowest density and tending toward zero near *N*_f_(*t*  + 1) =  *N*_f_(*t*). In GSD+TS and TSD+TS populations, the baseline embryonic survival (*a*) depended on temperature according to a normal distribution (Fig.1B).

Three normal TS curves were examined, which differed in their temperature of peak survival (23°C, 25°C, and 27°C) and the range of temperatures that produced non-zero baseline embryonic survival probabilities (16–31°C, 18–33°C, and 20–35°C, respectively) (Fig.1B). These curves were based on temperature ranges for embryonic survival reported for reptiles with GSD and TSD (Yntema and Mrosovsky 1982; Schwarzkopf and Brooks 1987; Steyermark and Spotila 2001; Birchard 2004; Harlow 2004; Hawkes et al. 2007).

### Male limitation (*B*)

Female fecundity *, B,* depended on the probability of fertilization of a female, Pr{ *fert}*, with a maximum value of *B*_max_:


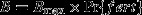
(3)

Pr{fert}was described as a function of adult sex ratio (ASR) following Rankin and Kokko (2007):


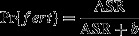
(4)

**Figure 2:**
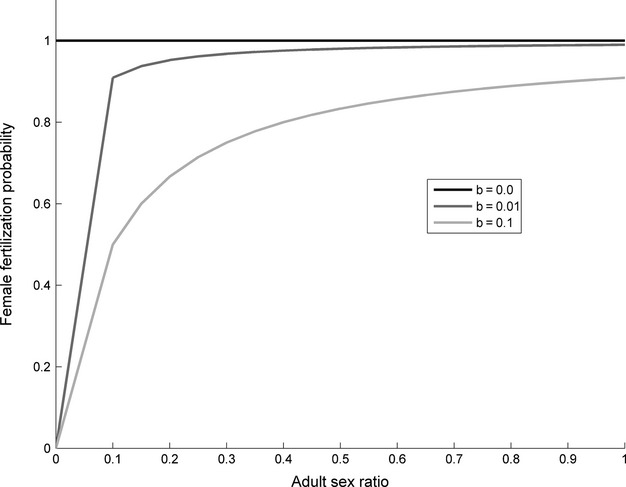
The female fertilization probability as a function of adult sex ratio (ASR). The different lines represent different sensitivities of fertilization probability to changes in the ASR (after Rankin and Kokko (2007)).

The shape parameter for equation 4, *b,* represents the relative strength of male limitation on female fecundity. There were three levels of male limitation on female fecundity considered, which varied from no limitation to strong limitation (Fig.2). When males were never limiting (*b*  = 0), female fecundity was always at its maximum (*B * =  *B*_max_). When males were limiting (*b*  > 0) the probability of female fertilization, and thus fecundity, decreased as the proportion of adult males in the population declined.

The adult sex ratio (ASR) was assumed to be equal to *p* (the CSR). Thus, we assume that males and females have the same mortality rates. This is a reasonable assumption in our model given that all offspring within a population experience the same temperature within and across years, and there is no immigration or emigration occurring with other populations. Under natural populations, this assumption would likely not hold (Girondot et al. 2004), and we would expect the population persistence to be enhanced or diminished depending on whether there is higher mortality in the rare or common sex, respectively.

## Results

### GSD

**Figure 3:**
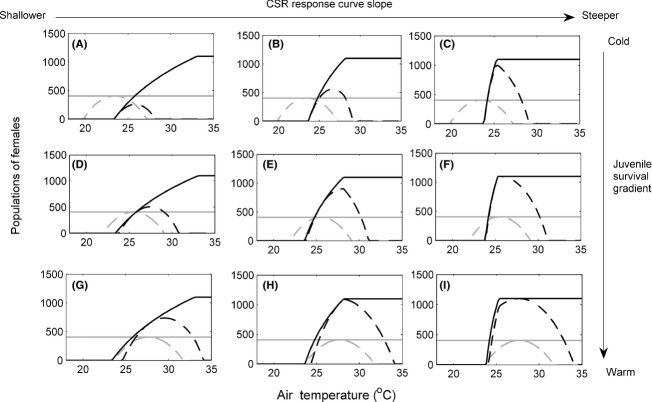
Populations of females for various combinations of temperature-dependent sex determination (TSD) (black lines) and genotypic sex determination (GSD) (gray lines) with (dashed lines) and without (solid lines) temperature-dependent embryonic survival (TS). Males do not limit female fecundity (*b * = * * 0). Cool embryonic survival gradient (A) to (C). Intermediate embryonic survival gradient (D) to (F). Hot embryonic survival gradient (G) to (I). TSD is shown with (A), (D), and (G) CSR response curve 2 (slope *β * = −0.069), (B), (E), and (H) CSR response curve 3 (slope *β*  = −0.147) and (C), (F), and (I) CSR response curve 4 (slope *β*  = −0.454).

**Figure 4:**
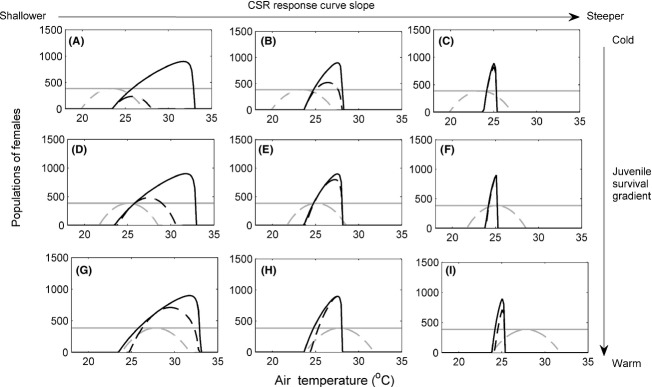
Populations of females for various combinations of temperature-dependent sex determination (TSD) (black lines) and genotypic sex determination (GSD) (gray lines) with (dashed lines) and without (solid lines) temperature-dependent embryonic survival (TS). Dashed curves for TSD species are not seen in panels (C) and (F) as TSD (no TS) (solid black line) completely overlaps with them. Moderate level of male limitation on female fecundity (*b * = * * 0.01). Cool embryonic survival gradient (A) to (C). Intermediate embryonic survival gradient (D) to (F). Hot embryonic survival gradient (G) to (I). TSD is shown with (A), (D), and (G) CSR response curve 2 (slope *β*  = −0.069), (B), (E) and (H) CSR response curve 3 (slope *β*  = −0.147) and (C), (F), and (I) CSR response curve 4 (slope *β*  = −0.454).

**Figure 5:**
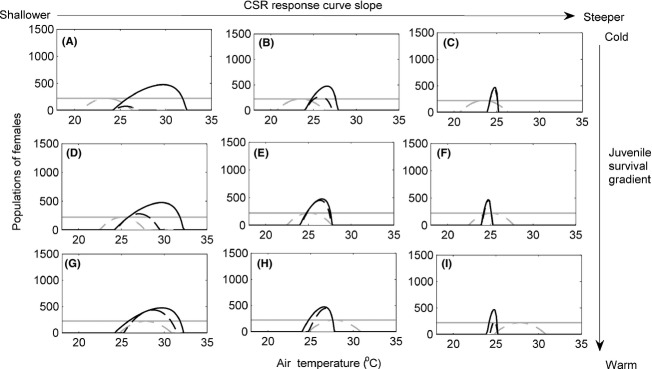
Populations of females for various combinations of temperature-dependent sex determination (TSD) (black lines) and genotypic sex determination (GSD) (gray lines) with (dashed lines) and without (solid lines) temperature-dependent embryonic survival (TS). Strong level of male limitation on female fecundity (*b * = * * 0.1). Cool embryonic survival gradient (A) to (C). Intermediate embryonic survival gradient (D) to (F). Hot embryonic survival gradient (G) to (I). Temperature-dependent sex determination (TSD) is shown with (A), (D), and (G) CSR response curve 2 (slope *β * = −0.069), (B), (E), and (H) CSR response curve 3 (slope *β * = −0.147) and (C), (F), and (I) CSR response curve 4 (slope *β * = −0.454).

For GSD(TS) populations, the independent effect of temperature-dependent survival on population persistence (range of temperatures at which populations persisted above size zero) and population sizes were clear (Table3, Figs3–5: solid gray lines vs. dashed gray lines). With TS, populations only persisted when baseline embryonic survival was greater than 0.011. By virtue of shape of the equation used to model male limitation, the highest level of male limitation actually reduced female fecundity, *B,* to be below maximum female fecundity, *B*_max_ even at an equal sex ratio, so population sizes differ according to strength of male limitation (Figs4 and 5).

**Table 3:** A description of the parameter space of baseline embryonic survival (*a*) and the proportion of male offspring (*p*) used to estimate the effects on population size (*N*)

Parameter	Definition	Range
Slope(*β*), *p* [ *T* ]	Relationship between *p* and air temperature	(0, −0.069, −0.147, −0.454)
*a* [ *T* ]	Relationship between *a* and air temperature	None and normal
*a*_max_ [ *T* ]	Peak of “ *a*_max_” occurs at three air temperatures (*T*)	23, 25, 27°C

### TSD, no male limitation (*b*  = 0)

If we examine the independent effect of temperature on sex ratio, we found that high air temperatures, which led to female-biased sex ratios, resulted in larger population sizes relative to the GSD populations (Fig.3). Conversely, low air temperatures led to male-biased sex ratios and extinction or smaller population sizes compared to GSD (Fig.3).

For TSD (no TS) populations, all CSR response curves resulted in strongly biased female CSRs and populations of the same large size at high enough temperatures (comparing solid black lines across columns in Fig.3). However, TSD populations with the steepest CSR response curve (curve 4) resulted in larger populations for a much smaller difference in temperature (within 1°C) and maximum population sizes at a lower air temperature (Figs3C, F, I). Hence, without male limitation, populations with steeper curves will grow faster in response to climatic warming compared to those with shallower curves.

We can examine the added effect of TS in a TSD species by comparing the solid and dashed black lines in each panel. Here, it is clear that reduced embryonic survival at extreme temperatures prevents large population sizes at warm, female-biased air temperatures (Fig.3). At the extreme hot temperatures, it leads to extinction. We can examine the interactive effect of TS and TSD by comparing the change between solid and dashed black lines across panels. Comparing the slope of the CSR curve (across columns), the CSR curve with the steepest slope (4) allowed populations of larger sizes than curves 2 or 3 even with TS (Fig.3 dashed lines, comparing columns), but had a very modest effect on the range of temperatures over which populations persisted. Thus, without male limitation, steeper CSR curves led to larger population sizes, but not greater ranges. This is because the populations reached sex ratios that are more female-biased at air temperatures that still had sufficient levels of embryonic survival to benefit from the extra production of females. That is, they recruited more females than GSD populations (Fig.3 gray lines), whereas the more shallow CSR curve (e.g. Fig.3A, D) only produced a heavily female-biased sex ratio at extreme temperatures where survival was so low that recruitment was equivalent or less than GSD populations.

The same pattern is apparent when comparing survival curves (comparing rows of Fig.3) – when peak survival coincides with female-biased sex ratios (bottom row), population size is greatly enhanced. One interesting difference is that, for the same CSR curve, survival curves that peaked at sex ratios closer to equity or male-biased not only led to reduced population sizes, but also the range of temperatures at which populations could persist. Thus, without male limitation, CSR slope influences population sizes but not range of temperatures of persistence, while coincidence of peak survival and female-biased sex ratios strongly influenced range of temperatures for persistence.

### TSD, male limitation (*b*  > 0)

If we examine the independent effect of male limitation (*b*  > 0) on population size and persistence in TSD species (Figs3, 4 and 5, comparing solid black lines of the same panels between figures), we found that high air temperatures, which led to female-biased sex ratios, no longer resulted in large population sizes. This is because male limitation (*b*  > 0) reduced fecundity to below *B*_max_, and large numbers of female offspring were no longer produced or recruited. Under even modest male limitation, TSD species have a very large reduction in the range of temperatures at which they can persist compared to GSD species (gray solid lines) and species with effectively no male limitation (Fig.3, black solid lines).

We can examine the interactive effects of male limitation and CSR slope by comparing how solid black lines change across columns between the zero (Fig.3), moderate (Fig.4), and strong (Fig.5) levels of male limitation. Whereas CSR slope influenced population size but not the range of temperatures for persistence when males are not limiting (Fig.3 black solid lines), male limitation produces the opposite result. In a world where low abundance of males can limit female fecundity, steeper CSR curves (right columns, Figs.4C, F, I and 5C, F, I) greatly reduced the range of temperatures at which populations can persist. Thus, with male limitation, CSR slope greatly influenced the range of temperatures of persistence. This is because as sex ratios became more female-biased with more modest increases in temperature, the probability of female fertilization decreased as males became rare, and hence, male limitation reduced fecundity to very low levels. The strong level of male limitation (Fig.5) reduced fecundity to lower levels for a given sex ratio than did the moderate level (Fig.4). As a consequence few female offspring were produced or recruited.

For TSD species with TS (dashed black lines, Figs3, 4 and 5), male limitation (*b*  > 0) overwhelmed the influence of embryonic survival in determining the range of temperatures of persistence as well as population sizes (Figs4C, F, I and 5C, F, I, right columns, dashed black lines). When comparing the importance of survival gradients under different levels of male limitation (black dashed line across rows compared across figures), it is clear that the enhanced range of persistence gained by peak survival coinciding with female-biased sex ratios under no male limitation (Fig.3G, H, I, bottom row) virtually disappears when males are limiting (Figs4G, H, I and 5G, H, I, bottom rows). The exception to this general result is with CSR response curve 2 (Figs4A, D, G and 5A, D, G, far left column). With a shallow CSR curve, we see that, regardless of the level of male limitation, having peak embryonic survival at warm temperatures increases the range of temperatures of population persistence. This is because warm air temperatures produce sex ratios that are closer to equality than for the other CSR curves, and thus, males are not rare, and *B* is still near *B*_max_. Hence, production and recruitment of female offspring across a wider range of temperatures was still possible (Fig.4A,D, G, far left column).

The results can be summarized by examining the 3-way interactive effect of TSD, male limitation, and TS (comparing the change between solid and dashed black lines across panels and figures). TSD led to extinction of cold populations compared to GSD populations across all scenarios (Fig.3, black solid vs. gray solid lines). Adding TS and male limitation (*b*  > 0) to populations with TSD reduced population sizes and resulted in more extinctions at warm air temperatures than when either were absent. While the effects of adding and varying TS were strong when males were not limiting (Fig.3, comparing solid and dashed black lines across rows), TSD with or without TS led to similar outcomes of population size and persistence when males were limiting (Figs4 and 5). The interaction between TSD and TS in a male-limited world was only important when the CSR curve was very shallow. In addition, the effects of male limitation (*b*  > 0) on population sizes and persistence were very similar irrespective of how female-biased the CSR was near peak embryonic survival when the CSR curve was steep (Figs4C, F, I and 5C, F, I, far right column, dashed lines). In all cases except when shallow CSR curves were associated with cold TS gradients (panel A in all figures), TSD populations had some range of temperatures at which their population sizes were greater than the maximum population sizes of the corresponding GSD populations.

## Discussion

The potential effects of biased sex ratios on the population dynamics of TSD species under warming climates have been highlighted extensively, yet quantitative predictions are rare (Hays et al. 2003; Hawkes et al. 2007, 2009; Poloczanska et al. 2009; Janzen 1994; Mitchell et al. 2008; Mitchell and Janzen 2010; Wapstra et al. 2009; Witt et al. 2010; Patino-Martinez et al. 2012). We still have little understanding of how important the strength of male limitation or climatically linked embryonic survival is for population persistence compared to biased sex ratios as climates warm. Our study demonstrated that the impacts of biased embryonic sex ratios on population persistence depend on how limiting male abundance is to female fecundity, how steep the relationship between cohort sex ratio and climate is, and what sex ratios are produced at peak survival. While it is intuitive that specifying male limitation and temperature-dependent embryonic survival will limit the capacity for population growth in female-biased populations, we also uncovered unpredictable results arising from the interactions of these factors.

We showed four key results. Firstly, cold temperatures (male-biased sex ratios) led to extinction in TSD populations compared with GSD populations in all instances. Populations with TSD were very sensitive to cool air temperatures, going extinct with a decrease of relatively few degrees in air temperature and at more than approximately 70% male offspring produced.

Secondly, population sizes and persistence in TSD species are very sensitive to variation in the strength of male limitation. In TSD species with (effectively) zero male limitation, higher air temperatures resulted in larger populations than in those with GSD and persistence across a wide range of temperatures. Strong female biases in both adult and embryonic sex ratios are thought to potentiate population persistence in warmer climates as large numbers of female offspring are produced and recruited (Freedberg and Taylor 2007; Mitchell and Janzen 2010; Doody and Moore 2011). Hence, warmer climates may be more beneficial for TSD species than GSD species.

However, these studies have not considered the impacts of male limitation on female fecundity and that TSD species will not benefit from warmer climates if males become limiting to female fecundity as they become rare. Our results show that male limitation at even a modest level in TSD populations located at warmer temperatures tended to negate the beneficial effects of the extra production of females due to female-biased sex ratios. Moreover, when the relationship between the cohort sex ratio and air temperature is similar to that seen for painted turtles and snow skinks (Wapstra et al. 2009; Schwanz et al. 2010), or even steeper, the limitation of males at warm temperatures overwhelms the impact of reduced embryonic survival. In essence, it does not matter if the warm air temperature leads to mortality of the common female hatchlings because they never would have succeeded in reproducing if they had lived.

Would the strong level of male limitation occur in TSD species? Males mate with multiple females in most reptiles (Broderick et al. 2001; Pearse et al. 2002; Uller and Olsson 2008), and female turtles are able to store viable sperm for up to 4 years, and hence, contact with males during that time may not be necessary for successful reproduction (Pearse et al. 2002). Male sea turtles are thought to reproduce more frequently than females and move considerable distances between assemblages of females in order to reproduce. Male limitation on female fecundity is thought to be low in these populations (Broderick et al. 2000; Poloczanska et al. 2009). However, it is almost impossible to obtain empirical data on male limitation for females in populations of marine turtles, given their wide-scale distribution and movements across oceans (Miller 1997; Wright et al. 2012). In contrast, in some lizard species with small home ranges and territoriality, declining male abundance may more strongly affect population persistence, if, for example, females are not able to store sperm, rates of reproduction are low, mobility is low due to landscape patchiness, or females are spatially dispersed when reproductively receptive. Male limitation on female fecundity is potentially high in such populations (Pearse et al. 2002). It may be possible to obtain empirical data on male limitation for those lizard species with small home ranges, given their distribution across relatively small areas, which may only be a few meters (Olsson and Shine 2003). This would be a highly useful avenue of research as a way of estimating the upper limits of male limitation likely in reptiles.

Our third key finding was that temperature-dependent embryonic survival is important in predicting population size and persistence only when males are effectively not limiting or when the relationship between cohort sex ratio and temperature is shallow (for example, as seen in loggerhead turtles, Hawkes et al. 2007). Under these scenarios, reduced embryonic survival at extreme temperatures prevents large population sizes at warm air temperatures with female-biased sex ratios. At extreme temperatures population extinctions occur. This is an important finding as the effect of reduced embryonic survival at warmer temperatures on populations that produce many females is less obvious than the effects of shortfalls in male abundance.

Finally, when temperature-dependent embryonic survival was important, the effect of varying the CSR at which peak embryonic survival occurs was very strong on population persistence in TSD species. In the absence of male limitation, the co-occurrence of female-biased CSRs and peak embryonic survival facilitated population persistence across the widest range of temperatures. In such a scenario, female-biased populations may survive and even thrive during short-term temperature increases. When males were limiting but the CSR curve was shallow, survival that peaked at warm temperatures allowed populations to persist across a range of temperatures nearly as broad as for populations without an effect of temperature on survival.

Temperature-dependent sex determination reptiles may compensate for the effects of climate warming on offspring survival or sex ratios through altered nesting behavior (evolutionarily or plastically). These compensatory mechanisms include the following: changing nest site choice (Hays et al. 2003; Fuentes et al. 2011); shifting nests to cooler microhabitats (Doody and Moore 2011); or shifting nesting to cooler months (timing) (Hays et al. 2003; Weishampel et al. 2004). Earlier nesting has been observed in several species in response to climate warming (Weishampel et al. 2004; Schwanz and Janzen 2008; Telemeco et al. 2009). Nesting plasticity may prevent increases in nest temperature that would otherwise reduce juvenile survival or generate imbalances in primary sex ratios. However, several studies (Morjan 2003; Schwanz and Janzen 2008; Wapstra et al. 2009; Telemeco et al. 2013) have suggested that phenotypic plasticity in female behavior in response to rising temperatures is not sufficient to ameliorate the effects of climate warming. Furthermore, a recent study has demonstrated that local adaptation by TSD reptiles to climate is not always possible (Harts et al., in press^2014^).

The collection of data for the CSR response curve, climatically linked embryonic survival, and male limitation are important research priorities as they can assist in understanding and predicting the size and viability of future reptile populations as climates warm. For example, more data needs to be collected on CSR and air temperatures to assess whether the CSR response curve can be consistently and effectively described in a similar manner to the TSD reaction norm (Hulin et al. 2009). Male limitation on female fecundity is an essential parameter on which data should be gathered in TSD reptiles. For example, there is already considerable available data on biased primary sex ratios (Janzen 1994; Mitchell and Janzen 2010), but there have been no formal analyses of the effects of male shortages on population persistence. Embryonic survival is also very important, as very little is known about at which temperatures eggs successfully incubate in many natural reptile populations or the thermal limits of embryonic survival. Hence, a large amount of empirical data are needed to test and validate our model. Our model will be an effective tool for empiricists and conservation managers in estimating the underlying population dynamics of TSD populations tending toward extreme female bias in a changing climate.
